# TMM@: a web application for the analysis of transmembrane helix mobility

**DOI:** 10.1186/1471-2105-8-232

**Published:** 2007-07-02

**Authors:** Lars Skjaerven, Inge Jonassen, Nathalie Reuter

**Affiliations:** 1 Computational Biology Unit, Bergen Center for Computational Science, University of Bergen, Thormøhlensgt.55, N-5008 Bergen, Norway; 2 Department of Informatics, University of Bergen, Thormøhlensgt 55, N-5008 Bergen, Norway; 3 Department of Biomedicine, University of Bergen, N-5009 Bergen, Norway

## Abstract

**Background:**

To understand the mechanism by which a protein transmits a signal through the cell membrane, an understanding of the flexibility of its transmembrane (TM) region is essential. Normal Mode Analysis (NMA) has become the method of choice to investigate the slowest motions in macromolecular systems. It has been widely used to study transmembrane channels and pumps. It relies on the hypothesis that the vibrational normal modes having the lowest frequencies (also named soft modes) describe the largest movements in a protein and are the ones that are functionally relevant. In particular NMA can be used to study dynamics of TM regions, but no tool making this approach available for non-experts, has been available so far.

**Results:**

We developed the web-application TMM@ (TransMembrane α-helical Mobility analyzer). It uses NMA to characterize the propensity of transmembrane α-helices to be displaced. Starting from a structure file at the PDB format, the server computes the normal modes of the protein and identifies which helices in the bundle are the most mobile. Each analysis is performed independently from the others and results can be visualized using only a web browser. No additional plug-in or software is required. For users who would like to further analyze the output data with their favourite software, raw results can also be downloaded.

**Conclusion:**

We built a novel and unique tool, TMM@, to study the mobility of transmembrane α-helices. The tool can be applied to for example membrane transporters and provides biologists studying transmembrane proteins with an approach to investigate which α-helices are likely to undergo the largest displacements, and hence which helices are most likely to be involved in the transportation of molecules in and out of the cell.

## I. Background

α-helical transmembrane (TM) proteins represent approximately 20–30% of all open reading-frames in the genome of complex organisms. They are involved in many biological processes such as sight, smell, muscle contraction, photosynthesis, *etc *[1]. Their signalling function is most often achieved by movements of the helices constituting the transmembrane bundle; the movements can be of different nature, involving the whole bundle like in the case of the mechanosensitive channel [2] or individual helices displacements such as those accomplished by the Ca-ATPase to transport calcium ions through the sarcoplasmic reticulum membrane [3]. Even though the structural changes involved are now pretty well understood for these two proteins, there are still many for which the available structural information is not sufficient to understand the mechanism(s) by which signals are transmitted along the transmembrane region.

Molecular modeling is the approach of choice to study protein dynamics. Molecular Dynamics (MD) is perhaps the most widely used technique but it is computer demanding and simulations of slow protein dynamics still take months even on high-performance computers. Approximate Normal Mode Analysis (NMA) is the method of choice to investigate the slowest motions in macromolecular systems [4]. Because of its very modest requirements in terms of computer power, it is especially useful for large biomolecular assemblies. It has been shown for many proteins that the vibrational normal modes having the lowest frequencies describe the largest movements in a protein and are the ones that are functionally relevant [4-9]. In recent years, it has become available to non-specialists thanks to the development of several web applications enabling traditional NMA approaches [10-15] for the study of large amplitude movements of protein domains.

NMA has proven useful in identifying mobile helices in TM bundles and describing, for example, the structural modifications accompanying the transport of calcium by the Ca-ATPase or the opening/closing mechanism of the mechanosensitive channel (MscL) [14,16,17]. Here we present a novel unique web application, the TransMembrane α-helical Movement Analyzer (TMM@) that analyzes the mobility of α-helices in transmembrane bundles. TMM@ performs a calculation of the normal modes and analyzes the displacement of the TM α-helices by calculating the overlap between the modes and displacement vectors describing movements with relevance to the transport function. The outcome is a ranking of the TM helices according to their mobility. For proteins where it is believed that the transport function involves helices movements, the characterization of their mobility will help elucidating the way the signal is transmitted through the cell membrane [18].

## II. Implementation

The architecture of TMM@ is similar to that of WEBnm@ [13]. The web-interface is written using the DTML language of the Zope [19] webserver. The analysis core, written in Python, runs under the BIAZ application server [20]. The calculation of the normal modes and the analysis of the displacements of the helices are implemented in Python and make extensive use of MMTK [21]. The normal modes are calculated on our server (AMD Opteron, 2 core, 2.4 Ghz, 8GB ram). The calculation of the whole set of normal modes for a protein of 1000 residues (i.e. 3000 modes) is performed in about 10 minutes. Overlap plots are generated using the R [22] package and the Rpy library [23]. We have embedded Jmol [24], an interactive molecular viewer (Java applet), into the web page to visualize the protein. VMD [25] (1.8.1) is used to produce VMD state files which can be downloaded if a more detailed visualization is preferred.

### 1. Normal modes calculations

A normal mode analysis (NMA) consists of the diagonalization of the matrix of the second derivatives of the energy with respect to the displacements of the atoms, in mass-weighted coordinates (Hessian matrix). The eigenvectors of the Hessian matrix are the normal modes, and the associated eigenvalues are the squares of the associated frequencies. We use the approximate normal modes calculation method developed by Hinsen [26] and implemented in the MMTK package [21]. This method represents the low-frequency domain motions very well at negligible computational cost. The force field used has been described in reference [27]. It uses only the Cα atoms of the protein; each atom is assigned the weight of the whole residue it represents.

Briefly, the functional form of the force field is

U(R1,...,RN)=∑all pairs i,j V(Ri−Rj)
 MathType@MTEF@5@5@+=feaafiart1ev1aaatCvAUfKttLearuWrP9MDH5MBPbIqV92AaeXatLxBI9gBaebbnrfifHhDYfgasaacH8akY=wiFfYdH8Gipec8Eeeu0xXdbba9frFj0=OqFfea0dXdd9vqai=hGuQ8kuc9pgc9s8qqaq=dirpe0xb9q8qiLsFr0=vr0=vr0dc8meaabaqaciaacaGaaeqabaqabeGadaaakeaacqqGvbqvdaqadaqaaiabbkfasnaaBaaaleaacqaIXaqmaeqaaOGaemilaWIaemOla4IaemOla4IaemOla4IaemilaWIaeeOuai1aaSbaaSqaaiabb6eaobqabaaakiaawIcacaGLPaaacqGH9aqpdaaeqbqaaiabbccaGiabbAfawnaabmaabaGaeeOuai1aaSbaaSqaaiabbMgaPbqabaGccqGHsislcqqGsbGudaWgaaWcbaGaeeOAaOgabeaaaOGaayjkaiaawMcaaaWcbaGaeeyyaeMaeeiBaWMaeeiBaWMaeeiiaaIaeeiCaaNaeeyyaeMaeeyAaKMaeeOCaiNaee4CamNaeeiiaaIaeeyAaKMaeiilaWIaeeOAaOgabeqdcqGHris5aaaa@55C5@

V (r) is the harmonic pair potential describing the interaction between the Cα atoms:

V(r)=k(|Rij(0)|) ( |r|−  |Rij0|) 2
 MathType@MTEF@5@5@+=feaafiart1ev1aaatCvAUfKttLearuWrP9MDH5MBPbIqV92AaeXatLxBI9gBaebbnrfifHhDYfgasaacH8akY=wiFfYdH8Gipec8Eeeu0xXdbba9frFj0=OqFfea0dXdd9vqai=hGuQ8kuc9pgc9s8qqaq=dirpe0xb9q8qiLsFr0=vr0=vr0dc8meaabaqaciaacaGaaeqabaqabeGadaaakeaacqWGwbGvcqGGOaakcqWGYbGCcqGGPaqkcqGH9aqpcqWGRbWAdaqadaqaamaaemaabaGaemOuai1aa0baaSqaaiabdMgaPjabdQgaQbqaamaabmaabaGaeGimaadacaGLOaGaayzkaaaaaaGccaGLhWUaayjcSdaacaGLOaGaayzkaaGaeeiiaaYaaeWaaeaacqqGGaaidaabdaqaaiabdkhaYbGaay5bSlaawIa7aiabgkHiTiabbccaGiabbccaGmaaemaabaGaemOuai1aa0baaSqaaiabdMgaPjabdQgaQbqaaiabicdaWaaaaOGaay5bSlaawIa7aaGaayjkaiaawMcaamaaCaaaleqabaGaeeiiaaIaeGOmaidaaaaa@52D6@

where Rij(0)
 MathType@MTEF@5@5@+=feaafiart1ev1aaatCvAUfKttLearuWrP9MDH5MBPbIqV92AaeXatLxBI9gBaebbnrfifHhDYfgasaacH8akY=wiFfYdH8Gipec8Eeeu0xXdbba9frFj0=OqFfea0dXdd9vqai=hGuQ8kuc9pgc9s8qqaq=dirpe0xb9q8qiLsFr0=vr0=vr0dc8meaabaqaciaacaGaaeqabaqabeGadaaakeaacqqGsbGudaqhaaWcbaGaeeyAaKMaeeOAaOgabaWaaeWaaeaacqaIWaamaiaawIcacaGLPaaaaaaaaa@332F@ is the pair distance vector (R_i _- R_j_) in the input configuration and k is the pair force constant:

k(r)={8.6×105kJ mol−1nm−3.r−2.39×105kJ mol−1nm−2for r<0.4 nm128 kJ nm4mol−1.r−6for r≥0.4 nm
 MathType@MTEF@5@5@+=feaafiart1ev1aaatCvAUfKttLearuWrP9MDH5MBPbIqV92AaeXatLxBI9gBaebbnrfifHhDYfgasaacH8akY=wiFfYdH8Gipec8Eeeu0xXdbba9frFj0=OqFfea0dXdd9vqai=hGuQ8kuc9pgc9s8qqaq=dirpe0xb9q8qiLsFr0=vr0=vr0dc8meaabaqaciaacaGaaeqabaqabeGadaaakeaacqqGRbWAcqGGOaakcqqGYbGCcqGGPaqkcqGH9aqpdaGabeqaauaabaqaciaaaeaacqaI4aaocqGGUaGlcqaI2aGncqGHxdaTcqaIXaqmcqaIWaamdaahaaWcbeqaaiabiwda1aaakiabbUgaRjabbQeakjabbccaGiabb2gaTjabb+gaVjabbYgaSnaaCaaaleqabaGaeyOeI0IaeGymaedaaOGaeeOBa4MaeeyBa02aaWbaaSqabeaacqGHsislcqaIZaWmaaGccqGGUaGlcqqGYbGCcqGHsislcqaIYaGmcqGGUaGlcqaIZaWmcqaI5aqocqGHxdaTcqaIXaqmcqaIWaamdaahaaWcbeqaaiabiwda1aaakiabbUgaRjabbQeakjabbccaGiabb2gaTjabb+gaVjabbYgaSnaaCaaaleqabaGaeyOeI0IaeGymaedaaOGaeeOBa4MaeeyBa02aaWbaaSqabeaacqGHsislcqaIYaGmaaaakeaacqqGMbGzcqqGVbWBcqqGYbGCcqqGGaaicqqGYbGCcqGH8aapcqaIWaamcqGGUaGlcqaI0aancqqGGaaicqqGUbGBcqqGTbqBaeaacqaIXaqmcqaIYaGmcqaI4aaocqqGGaaicqqGRbWAcqqGkbGscqqGGaaicqqGUbGBcqqGTbqBdaahaaWcbeqaaiabisda0aaakiabb2gaTjabb+gaVjabbYgaSnaaCaaaleqabaGaeyOeI0IaeGymaedaaOGaeiOla4IaeeOCai3aaWbaaSqabeaacqGHsislcqaI2aGnaaaakeaacqqGMbGzcqqGVbWBcqqGYbGCcqqGGaaicqqGYbGCcqGHLjYScqaIWaamcqGGUaGlcqaI0aancqqGGaaicqqGUbGBcqqGTbqBaaaacaGL7baaaaa@95BD@

### 2. Identification of trans-membrane α-helices bundle

TMM@ uses DSSP [28] and its own filter algorithm to produce a list of all α-helices present in the submitted protein structure and to identify the TM bundle. The filter algorithm makes use of the following structural properties: helix length, distance between helices, hydrophobicity, and the angle between helices. As the filter algorithm is based on empirical parameters, we recommend that each user review and correct if necessary the list suggested by TMM@.

### 3. Defining α-helical mobility

The projection of a normal mode vector onto a displacement vector defines the contribution of the normal mode to the given displacement. In TMM@ we define four different movements of relevance for the transport function: (i) rotation and (ii) translation of individual helices around and along their axis, respectively, (iii) slide of the α-helices perpendicular to the helix axis towards/away from the centre of the bundle, and (iv) tilt of helices perpendicular to the helix axis away from the centre of bundle, and (v) rotation of the helices around the bundle axis. The axis of a α-helix is defined as the principal axis of inertia of the Cα-atoms of the amino acids forming the helix, the axis of the bundle is defined as the principal axis of inertia of the Cα atoms of all helices in the bundle. The rotation vector on each Cα-atom of the α-helix is calculated as the cross-product between a unit vector collinear to the helix axis and the distance vector between the Cα-atom and the centre of mass of the α-helix. The translation vectors of the α-helices have a component for each Cα-atom, collinear to the axis. The rotation of the bundle is defined by the cross product between the axis of the bundle and the distance vector between the bundle centre and the helix centre. The slide vector is the cross product between the bundle rotation vector and the helix axis. The tilt vector is calculated in the same way as the slide vector, but with decreasing magnitude for residues closer to the centre of the helix, and opposite direction on the other side of the centre. Hence, we tilt the helix around the centre of the helix, directly away and towards the bundle axis.

The projections are defined by

*p*_*i *_= **d**·*e*_*i*_

where ***e***_***i ***_is the normal mode vector of mode *i*, and **d **is the displacement vector (i.e. rotation, translation, slide or tilt of individual helices, bundle rotation). This satisfies the relation

**Figure 1:**
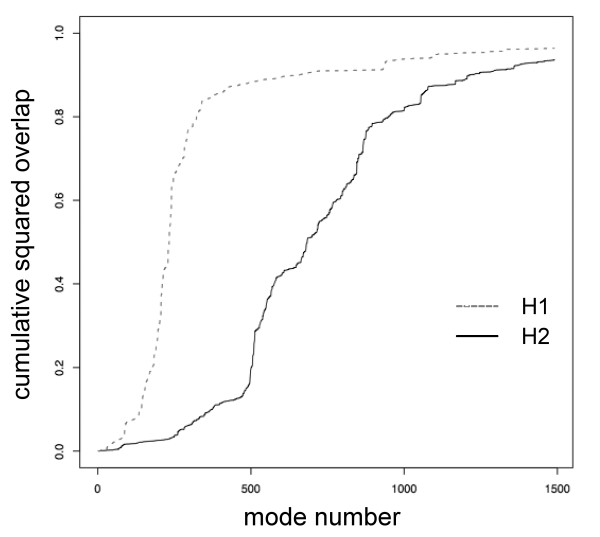
**Cumulative squared overlap**. Example plot for the rotation of two helices (named H1 and H2) of the calcium pump around their own axis.

∑i=13Npi2=1
 MathType@MTEF@5@5@+=feaafiart1ev1aaatCvAUfKttLearuWrP9MDH5MBPbIqV92AaeXatLxBI9gBaebbnrfifHhDYfgasaacH8akY=wiFfYdH8Gipec8Eeeu0xXdbba9frFj0=OqFfea0dXdd9vqai=hGuQ8kuc9pgc9s8qqaq=dirpe0xb9q8qiLsFr0=vr0=vr0dc8meaabaqaciaacaGaaeqabaqabeGadaaakeaadaaeWbqaaiabdchaWnaaDaaaleaacqWGPbqAaeaacqaIYaGmaaaabaGaemyAaKMaeyypa0JaeGymaedabaGaeG4mamJaemOta4eaniabggHiLdGccqGH9aqpcqaIXaqmaaa@3A30@

because the normal mode vectors form a basis of configuration space (N is the number of atoms). Thus *p*_i_^2 ^is interpreted as the contribution of mode *i *to the motion described by **d**. For each helix, the calculation of the cumulative overlap of one given displacement vector and all modes thus yields a curve that increases from 0 to 1 (y axis) when it is computed over all modes (on the x axis). The modes are ranked following increasing frequencies. Frequency is inversely proportional to amplitude. If α-helix H1 shows a plot (e.g. grey dotted line on Fig. 1) that reaches an asymptotic behaviour for fewer modes than the plot of another helix H2 (e.g. black plain line on Fig. 1), it means that the movement of H1 following **d **is of higher amplitude than H2 following **d**. As a consequence H1 is considered to be more mobile than H2. This is illustrated on Figure 1 where **d **is the rotation of each helix around its own axis.

Song et al[29] recently described a new method to evaluate the overlap between a set of normal modes and a given transconformation. They point out that the global conformation displacement is a finite motion, while the normal mode motions are infinitesimal motions. Therefore, for large conformational difference, the global direction may have little to do with the initial direction. They thus propose to use an infinitesimal version of the transconformation instead of the global transconformation, and show that it is more reliable than the usual difference vector, especially when the displacement is large. However, our program is meant to evaluate the contribution of the modes to helices displacements that we define ourselves, using only one structure. We thus avoid the problems mentioned by Song et al.

## III. Results and Discussion

TMM@ can be used by anybody with access to an internet browser; all results are presented within the webpages of TMM@ and no additional programs are needed on the computer of the user. The user starts by defining which structure is to be analyzed; from the main page, he/she is offered the possibility to upload his/her own protein structure file (in the Protein Data Bank format) or specify a PDB code (a local copy of the PDB database is maintained on our server). A third field allows the user to enter a job ID to continue working on a previously started calculation. Pressing the *submit *button will launch the calculation of the normal modes. Then TMM@ identifies all α-helices present in the protein and lists them on the next page where it also suggests which ones are TM helices. The user is offered the possibility to correct the definition of the TM α-helices with the aid of a Jmol applet. Alternatively the user can download a vmd state file [25] for a more detailed visualization of the predicted TM helices. For each TM helix in the bundle approved by the user, TMM@ defines the displacement vectors and calculates the overlap between each of them and the set of normal modes. The overlaps are then plotted using the R package and the Rpy library. The cumulative squared overlaps are plotted against mode numbers, four plots are drawn (available in the PDF and PNG formats); one for each type of displacement. In addition, the user can retrieve the raw results in a text file. Each curve on the plot corresponds to a TM α-helix, which is identified with a unique identifier, colour and line type. Displaying all helices on the same plot helps comparing their mobility. It is reasonable to believe that the most mobile helices will be involved in the structural modifications accompanying, for example, the transport of a molecule or ion through a tight TM bundle. In many cases, the transport function is indeed a dynamical process during which the protein undergoes structural rearrangements.

**Figure 2:**
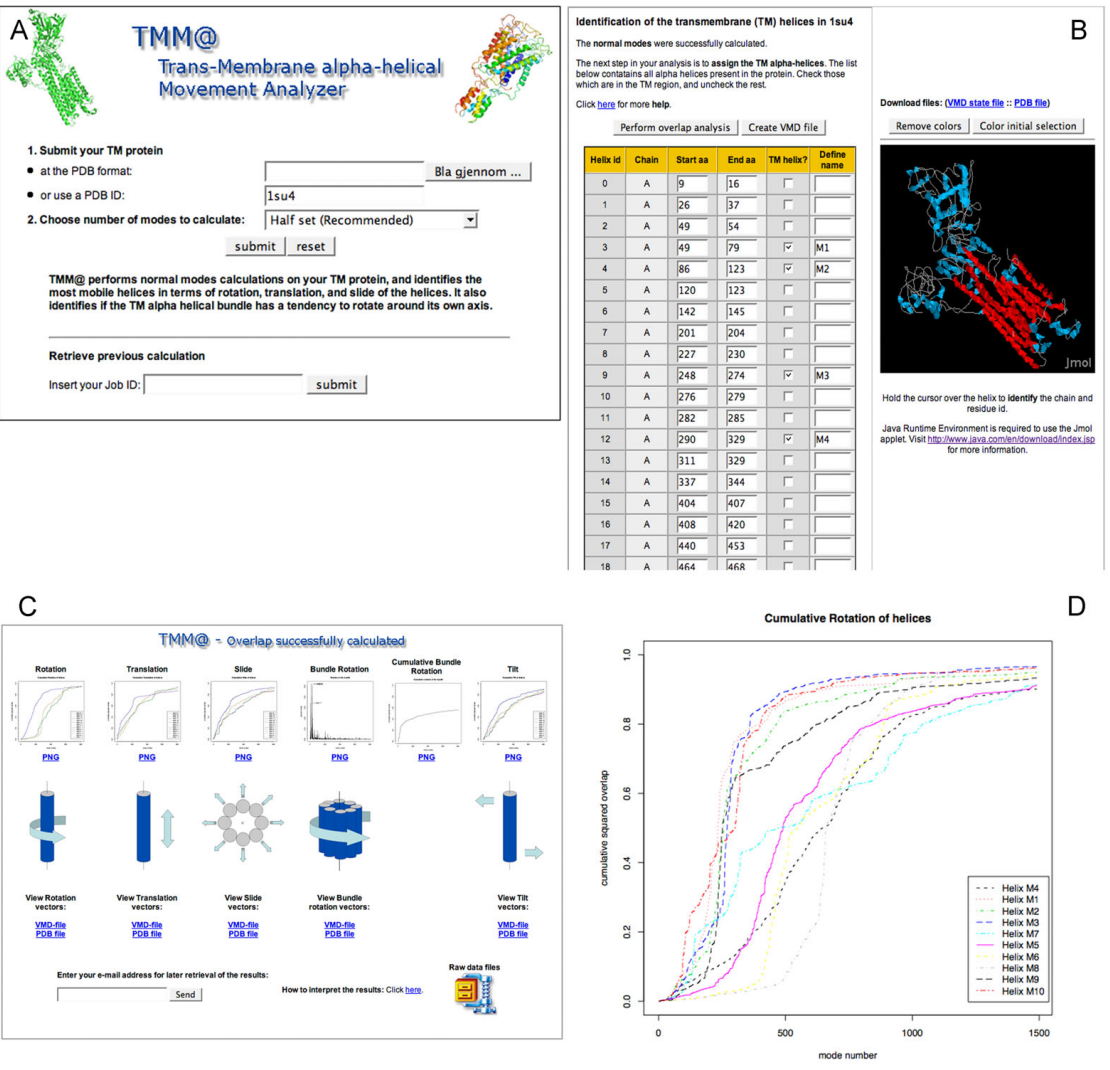
**Snapshots of an example calculation on the E1Ca form the SERCA I Ca-ATPase (1su4)**. **a**. The main page of TMM@ is a form where users can input a structure file in the PDB format. **b**. Identification of the TM α-helices (Cf. Table 1). **c**. Overview of the results of the overlap analyses. The user can download either ready-made plots (pdf or png formats) or an archive containing all the raw data and draw his own plots. **d**. The overlap plot for the rotation displacement of each helix around its own axis.

**Figure 3:**
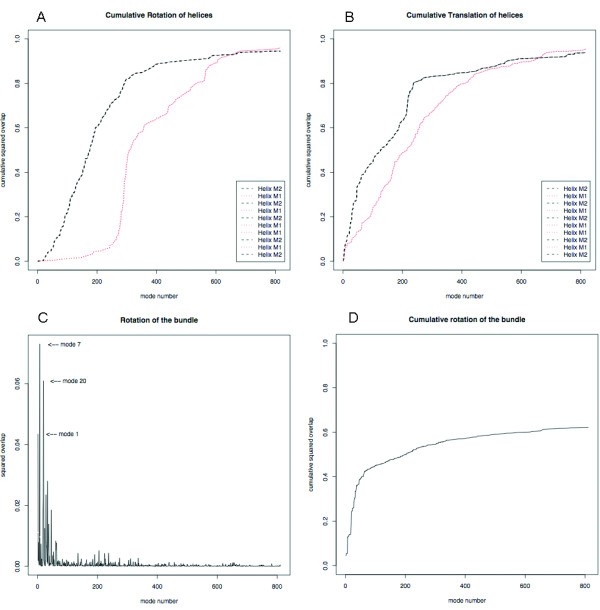
**Cumulative overlap for the helices of MscL**. Plots for the rotation (a) and translation (b) displacements of each helix around and along its own axis, respectively. Overlap (c) and cumulative overlap (d) of the rotation of the TM bundle around its axis.

We have tested TMM@ on more than 20 transmembrane proteins taken from the PDB, and representing different families. In what follows, we describe two examples and use them to illustrate the different steps performed by TMM@: the SERCA1 calcium ATPase and the mechanosensitive channel (MscL). Snapshots of the application are given in Figure 2 and Figure 3, respectively. The calcium pump (Ca-ATPase) transports calcium from the cytoplasmic side (outside cell) to the lumenal side (inside cell). There is however no obvious channel in the protein leading to the lumenal side and it has been shown that the ion transport implies movements of the α-helices. After uploading the x-ray structure (1su4) of the E1Ca form of Ca-ATPase (Figure 2a) the normal modes will be computed. The filter algorithm outputs a list of 45 α-helices, in which 13 of these are suggested to be in the TM region (Figure 2b). However, since we know that only 10 of them are TM helices, we wish to correct the definition given by TMM@. The Jmol applet provided on the page aids in this work. The predicted TM helices of the Ca-ATPase are listed in Table 1. The next step is to submit the list of TM helices so that the overlap calculation will be performed, resulting in 5 plots (Figure 2c and 2d) described above. The overlap plot for the rotation of each helix around its individual axis (Figure 2d) shows that less than 300 modes are enough to describe 60% of the rotation of helices 3, 4 and 9 (M1-M3). Similarly, 60% of the rotation of helices 41 and 43 (M9 and M10) can be described with only 300 modes. Conversely, over 600 modes are needed to describe 60% of the rotation for helices 12, 31, 32, 34, 38 (M4 to M8). This means that helices number M1 to M3 can undergo larger amplitude displacements than helices M4 to M8. This leads to the hypothesis that they might play a role in the uptake/release of calcium ions since it is known that the ion transport requires displacements of the TM α-helices. This result is in agreement with the available x-ray structures of the calcium pump and in particular ref. [30] where Toyoshima et al. describe movements of M1 to M3, and in particular that M2 and M1 are pulled towards the cytoplasm by one and two turns of a α-helix, respectively. These complex movements are a combination of the rotation and translation around and along α-helical axes that TMM@ investigates opportunely.

**Table 1:** 

**Helix id (TMM@)**	**first aa – last aa**	**Correction by user**	**Name**
3	55–79	48–79	M1
4	86–118	-	M2
9	248–274	-	M3
12	290–307	289–329	M4
17	440–453	Not in TM bundle	
25	629–640	Not in TM bundle	
31	740–782	-	M5
32	789–800	788–808	M6
34	831–857	-	M7
38	894–912	-	M8
41	931–950	-	M9
43	964–975	963–992	M10
44	976–992	Into helix 43	

**α-helices of the SERCA1 Ca-ATPase**. List of α-helices identified by TMM@ as being transmembrane helices (Cf. Figure 2b); first and last residues are given in the second column. Corrections to be made by the user are listed in the third column. Correspondence with the usual nomenclature for the calcium pump (M1 to M10) is given in the fourth column.

Figure 3 (a–d) shows the resulting overlap plots for MscL (1msl). As expected, our calculations reproduce the symmetry of the molecule, i.e. all M2 helices have the same mobility in rotation and translation (Fig. 3a and 3b) around/along their own axis, the same applies to M1 helices. Therefore the plots show superimposed lines for symmetrically equivalent helices. For a bundle without that type of symmetry, like in the Ca-ATPase which contains 10 TM helices, the corresponding plots should contain 10 distinct lines. TMM@ also identifies the iris-like movement described by others [2,16]. The plots on Figure 3c and 3d indeed reveal that a few low-frequency modes (modes #1, #7 and #20) describe a rotation of the helices around the bundle axis. The same type of movement was identified for the calcium pump (Figure 2c and ref. [18]).

## IV. Conclusion

We have successfully implemented and developed a unique tool for analysing the mobility of *α*-helical TM segments in proteins. We have tested it on a number of TM α-helical proteins and have compared the results, whenever possible, with existing structural data. In these cases, the helices that TMM@ identifies as being the most mobile are known experimentally to be involved in the protein function. We thus believe that this approach has a strong predictive power.

Using normal mode calculations as the basis of the tool makes it reliable, robust and fast. Providing the service in a user friendly web interface will make it easy to use, even for non-specialists. It thus provides biologists studying transmembrane proteins a unique tool for determining which helices undergo the largest displacements, and hence which might be involved in the transportation of molecules in and out of the cell. Considering that trans-membrane proteins are of vital importance for cell life, TMM@ might be a tool with great value. TMM@ is available from the website of the Norwegian Bioinformatics Platform .

## Availability and requirements

Project name: TMM@

Project home page: 

Operating system: Platform independent; tested on Windows XP (Firefox 2.0, MS Explorer 6.0 and 7.0), Mac OSX (Firefox 2.0, Safari 2.0), CentOS and Ubuntu Linux (Firefox 2.0, Opera 9.0)

Programming language: Python

Other requirements: Java

## Authors' contributions

LS designed TMM@ and wrote the code. IJ was involved in the planning of TMM@ and contributed to the writing of the manuscript. NR supervised the project, and edited the manuscript. This work is a truly collaborative effort of all three authors. All authors read and approved the final manuscript.
